# The Doctor in Politics

**Published:** 1908-01

**Authors:** 


### The Doctor in Politics.

PROMPTED by the fact that seven doctors were chosen mayors of as many cities in New Jersey at the recent election, the *Evening Mail* (New York) makes appropriate comment under the above title, asseverating that the profession of medicine is coming into greater prominence in public life than ever before in this country. The *Mail* remarks, inter alia, that "In Dr. Leonard Wood the American army has a possible future commander-in-chief. Dr. Bensel has given New York a better administration of the department of street cleaning than it has had since Col. Waring's day. His predecessor, Major Woodbury, was a surgeon. In Dr. Gallinger, of New Hampshire, the medical profession has at least one representative in the federal Senate."

It may be added with appropriateness that in the House of Representatives, Dr. Hiram R. Burton, of Delaware, is an able representative of the medical profession and one who does not fail on all proper occasions to advocate the advancement of the guild of medicine. In recognition his efforts in this direction he was elected one of the vice-presidents of the American Medical Association during its meeting at Atlantic City last June.

In nearly every city or larger town one or more physicians hold elective office. In Buffalo, Dr. H. H. Bingham was recently elected a member of the city council, an office he has held heretofore, we believe. Dr. Eugene Beach, of Gloversville, has been mayor of that city for several years. Dr. E. F. Brush, of Mount Vernon, has held the office of mayor more than once, and his last election was secured on an independent ticket. He triumphed over the candidates of both the great parties and has popularised his administration by securing many needed reforms.

In Europe it is not uncommon for medical men to hold high place in the councils and courts of even some of the greater nations. Virchow, it will be remembered, was a leader in the Reichstag for many years; while a later example of the doctors' success in politics is in the person of M. Clemenceau, the great French premier, who was educated as a physician and practised his profession in his earlier years. It is a matter of general knowledge that the great majority of the delegates to the Central American peace congress at Washington are physicians by education.

Referring again to the *Mail's* article, it says: "To the tasks of political life the doctor can bring his skill in diagnosis, his knowledge of pathological conditions, the ability to jolly the individual which is half his curative art, and a wide acquaintance with the people and needs of his district. He should make a good legislator. Every successful physician, indeed, is something of a politician in the treatment of his patients. Why should not the converse be true?"

We think this a just comment and one that gives forceful reasons why physicians may serve their fellow-citizens wisely in public life. A good physician should make a good officer, but we imagine many such an one would hesitate to take office that would entirely separate him from his professional work for any considerable length of time. However, it ought not to be difficult for a physician well-grounded in his profession to recover his lost or diminished practice within a reasonable time after his return to private life.

Dr. Charles A. L. Reed, of Cincinnati, has dealt extensively with this subject in a paper published some months ago in the *Journal of the American Medical Association.* The statistics given by Dr. Reed are interesting and the paper may be read with profit by all who wish to be well informed on this phase of public life.

Returning to the results of the election in New Jersey, which prompted us to these remarks, we think it fair to conclude that in the seven cities that have elected physicians as mayors—namely, Atlantic Highlands, Frenchtown, Paterson, Rahway, Summit, Trenton, and Washington,—there will be improved sanitation and rigid administration of public health laws and ordinances. If this should prove true it will be something for which to be thankful.

